# Analysis of Universal Health Coverage and Equity on Health Care in Kenya

**DOI:** 10.5539/gjhs.v8n7p218

**Published:** 2015-12-16

**Authors:** Timothy Chrispinus Okech, Steve Ltumbesi Lelegwe

**Affiliations:** 1 Associate Professor of Economics, United States International University, Africa; 2 University of Nairobi, Nairobi, Kenya

**Keywords:** health equity, health infrastructure, human resources for health, solidarity

## Abstract

Kenya has made progress towards universal health coverage as evidenced in the various policy initiatives and reforms that have been implemented in the country since independence. The purpose of this analysis was to critically review the various initiatives that the government of Kenya has over the years initiated towards the realization of Universal Health Care (UHC) and how this has impacted on health equity. The paper relied heavly on secondary sources of information although primary data data was collected. Whereas secondary data was largely collected through critical review of policy documents and commissioned studies by the Ministry of Health and development partners, primary data was collected through interviews with various stakeholders involved in UHC including policy makers, implementers, researchers and health service providers. Key findings include commitment towards UHC; minimal solidarity in health care financing; cases of dysfunctionalilty of health care system; minimal opportunities for continuous medical training; quality concerns in terms of stock-outs of drugs and other medical supplies, dilapidated health infrastructure and inadequqte number of health workers. Other findings include governance concerns at NHIF coupled with, high operational costs, low capitation, fraud at facility levels, low pay out ratio, accreditation of facilities, and narrowness of the benefit package, among others. In lieu of these, various recommendations have been suggested. Among these include promotion of solidarty in health care financing that are reliable and economical in collecting; political will to enhance commitment towards devolution of health care, engagement of various stakeholders at both county and national government in fast tracking the enactment of Health Act; investment in health infrastructure and training of human resources; revamping NHIF into a full-fledged social health insurance scheme, and enhancing capacity of NHIF human resources, enhanced awareness amongst members, enhanced benefit package among other recommendations.

## 1. Introduction

### 1.1 Background

**Figure 1:**
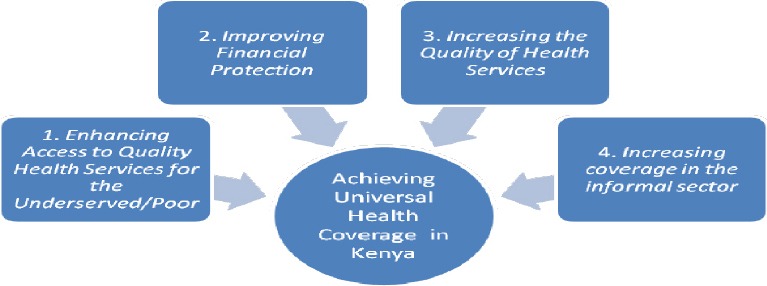
Universal Health Coverage

Universal health coverage ensures that all people can use the promotive, preventive, curative, rehabilitative and palliative health services they need, of sufficient quality to be effective, while also ensuring that the use of these services does not expose the user to financial hardship has continued to dominate demate in health care. This continues to attract the attention of many stakeholders including governments. This is because it embodies three related objectives namely equity in access to health services-those who need the services should get them, not only those who can pay for them; that the quality of health services is good enough to improve the health of those receiving services; and finally financial risk protection which aims at ensuring that the cost of using care does not put people at risk of financial hardship. Figure 1 provides the summary of universal health coverage.

Universal coverage brings the hope of better health and protection from poverty for hundreds of millions of people - especially those in the most vulnerable situations. Universal coverage is firmly based on the WHO constitution of 1948 declaring health a fundamental human right and on the Health for All agenda as stipulated in the Alma-Ata declaration of 1978. Four key elements are identified by World Health Organization (WHO) necessary towards the realization of universal coverage. One, a strong, efficient, well-run health system; two, a system for financing health services; three, access to essential medicines and technologies; and finally a sufficient capacity of well-trained, motivated health workers (WHO, 2010.

In Kenya, with support of various stakeholders, the government of Kenya has over the years since independence in 1963 initiated policy reforms and strategies earmarked towards universal health coverage. Some of these are outlined in various policy documents including Kenya Health Policy Framework (KHPF 1994–2010), Health Sector Strategic Plans, Vision 2030 (operationalized through the medium term expenditure framework of 2008-2012), the Constitution 2010, and finally, the Health Bill of 2015. Notably, the government recognized a high quality of life as a key pillar towards accelerating Kenya’s intentions of being a globally competitive and prosperous nation (GoK, 2008). Further the government provides a legal framework for ensuring a health care delivery system that is driven by the people while brindging the gap on geographical access by providing for a devolved system of governance (GoK, 2010). These initiatives can be argued are aimed towards universal health coverage for the populace in the country. In the draft Health Bill of 2015, the government has declared access to to reproductive health and emergency medical treatment as a right by all persons. This paper takes stock of these policies and reforms earmarked towards UHC and how they have impacted on health care especially on health equity issues in terms of utilization, access, quality care and protection against financial hardships. However, before enumerating these policies, the methodology utilized as well as a brief of the health situation and health services delivery are provided in that order.

### 1.2 Methodology

In order to obtain necessary information, both primary and secondary data were collected regarding health care reform initiatives and how these relate to universal health coverage. In terms of secondary data, a review of relevant literature on key policy initiatives aimed at universal coverage and how they have impacted on equity, access and financial protection. Secondary information was obtained from Ministry of Health official documents such as the draft Kenya National Health Sector Strategic Plan (KHSSP) III, draft Health Policy Framework, 2012–2030, draft Health Care Financing Strategy, and National government documents such as Vision 2030, Medium expenditure Framework (MTEF) paper, National Hospital Insurance fund documents (Manuals, statergic plans, aperational plans, among others), the Consitution and the Draft Health Bill, 2015. Additional data was also collected from relevant commissioned studies by Health Policy Initiatives, KFW, World Bank, Print and Mass media coverage, among others.

### 1.3 Health Situational

Kenya’s health indicators though showing a mixed trend since independence in 1963 to present, continue to lag behind those of the rest of world including sub-Saharan Africa (SSA). During the period, life expectancy rose from about 43.4 years (1960) to 62 years (1990), before declining and stabilizing at about 52 years (2006) and now estimated at 62 years (Male-60 years and female 65 years)(PRB, 2015). Infant mortality on the other hand dropped from 122 per 1,000 live births (1960) to 63 in 1990, before rising to 83 in the year 2000, followed by a drop to the current level of 39/1000 (PRB, 2015). The estimates for the under-five-year mortality rate over the same period were 204 per 1,000 live births, 93, 134 and 77 respectively currently estimated at 52/1000 (GoK, 2014). Finally, maternal mortality rates still remain high at 414 per 100,000 live births, 650 in 1990 and 1,000 in the year 2000 and finally 400/100,000 in 2015 (GoK, 2014; PRB, 2015). Evidently, these rates are far above the targets set for the MDGs for the country. Statistics shows that malaria account for almost half of morbidity in the country. It is ranked the third cause of death and an estimated 70% of the population is at risk of infection, with on average about 93 children dying every day from malaria infection.

### 1.4 The Health Services Delivery

Availability and comprehensiveness of health services offered at a health facility is critical in realizing UHC as reitereated by World Health Organization. As observed by WHO, this depends partially on the number and quality of health workers at facilities. Available statistics reveals that, there exist overall staff shortages with an estimated fifty thousands personnel, against an estimated minimum requirement of over seventy two thousands. This situation is likely to be worse given the recent exodus of health workers in the government facilities occasioned by the devolution of health care in 2013. Although concerted efforts have been made to increase the number of skilled health workers since independent, cases of skewness have been reported with many preferring urban areas as opposed to rural areas. This is partially attributed to difficulties in recruitment and retention of the workers. Reports show that this is due to incentives for locating in hard-to-reach places, general motivation and incentives, working conditions (including infrastructure and equipment), access to higher institution of learning for purposes of continous medical education (CME) for purposes of advancing in skills and career growth, dysfunctionality of human resource management at the devolved level with cases of low morale, disjointed promotions, salary differentials amongst workers in the same job group across counties, among others. This according to health workers professional associations is due to lack of schemes of work at the county level. This negates the provisons of World Health Organization whereby a sufficient capacity of well-trained and motivated health workers is key in realizing universal coverage.

Studies and media reports reveal that many public hospitals have dilapidated health infrastructure and have limited appropriate equipment thanks to the initiatives of the First Lady on “*zero”* tolerance in equipping facilities country wide. The available infrastrucuture has however continued to impact negatively on the care as well as the ability to retain some key health personnel especially, specialized health workers in the public service. Cases where for instance specialized doctors complained of underutilization of their skills have been experienced with many opting to join private practice or resigning to pursue further studies. If the situation is not addressed, in the end, patients are likely to be left with no option but to either seek services of less qualified health personnel or providers or alternative health care services whose quality may not be guarantered. Worse, others may seek services from private facilities which may be relatively expensive thereby negating the expected gains of financial risk protection currently being pursued under the enhanced National Hospital Insurance Scheme (NHIF). Similarly, cases of significant gaps in essential specialized care capacity exists forcing individuals to seek these services abroad again impacting the pursuit of financial protection. On the other hand, whereas the “*zero*” tolerance initiative are lauded, human capacity remain a concern as this seem not to be addressed accordingly by the two-tier of governments. In the end, the equipment may remain idle despite the efforts of the First Lady. Media reports are awash of cases misplaced investments in health care delivery at county level and limited focus on continuous medical education for purposes of upgrading on health technology considered key in UHC key ingredients in te realization of universal coverage.

In terms of provision of health care, the services are currently provided by both private and public health facilities (both County and National government). Private facilities include faith based facilities run by religious institutions and non-governmental organizations and private-for-profit providers. In a nutshell, the country exhibits a robust public/private mix in healthcare service providers. Due to inability of a large segment of the population to afford private sector healthcare services given the poverty levels, and the charges by the private facilities, over-utilization the public facilities has been witnessed. Whereas this is good, issues of quality and huge work load for the already stretched health workers are likely to be experienced.

## 2. Towards Universal Coverage and Health Equity

### 2.1 Introduction

The pursuit of UHC has been a critical focus for many health care providers including governments, multilateral institutions, NGOs as well as CBOs. The Kenya government with support from various stakeholders has continued to develop and implement national health policies and strategies aimed at enhancing access to quality and affordable health care. To realize this, the government with support from key sectoral partners has over the years continued to reform the health sector as a bold step towards universal coverage. These reforms are contained in the various policy documents including Health Policy Framework Paper, National Health Sector Strategic Plan I & II, Vision 2030 and the constitution as well as the draft Health bill 2015.

### 2.2 From Independent to Early 2000s

Upon attaining independence in 1963, the Government of Kenya (GoK) in recognized the pivotal role of health towards socioeconomic development, embarked on wider policy reforms aimed at enhancing access to quality care. A number of government policy documents and successive national development plans were developed wherein policies and strategies were mooted towards enhancing geographical access which then was limited to the whites who were the minority. As a result of these policies, health indicators such as infant and child mortality, and life expectancy started to improve (GoK, 2010). This was partily attributed to enhanced provision of primary health care (PHC) and continued training of skilled health workers in line with WHO guidelines. Similarly, the government expanded the training program for the various cadres of health personnel and health infrastructure in various parts of the country. This is critical since both infrastructure and health personnel are key ingredients as reiterated by World Health organization in 2010. Other initiatives included progressive elimination of fees in public health facilities as part of the strategy of increase access to health care. Overtime, the health sector in Kenya has operated in the context of a number of policy frameworks. In 1980’s the policy shift from purely government provided for care to cost sharing was followed by the 1993 institutional and structural reforms, and market orientation of the health services. It is however worth noting that during this period, there were a number of policy reversals with mixed equity issues. For instance, following the dwindling donor funding and the macroeconomic problems the country experienced, coupled with continuous surge in population, the health sector became too large for the government to handle single handed. To cope with this, the government introduced cost sharing in public health in *“toto”* though abandoned before reintroducing it a few years later. To caution the poor, and other vulnerable groups, the government with support from development partners, introduced a system of waivers and exemptions which was however riddled with implementation weaknesses with minimal realization of the intended objectives.

In the recognition of the role of a well run health system and financing play in contributing towards universal coverage, the government in 1994, developed the Health Policy Framework and a five-year National Health Sector Strategic Plan (NHSSP) of 1999-2004 wherein targets and processes driving the health sector development, as well as healthcare service delivery were articulated. For instance, reforms relating to the way the healthcare services were not only organized but also financed, delivered and evaluated were initaited. This was. In the document, equitable allocation of government resources to reduce disparities in health status and increased cost-effectiveness and efficiency of resource allocation and use were emphasized. This partially contributed towards the improvement inhealth outcomes especially infant, child and under-five mortality rates as summarized in Fugure 2.

**Figure 2:**
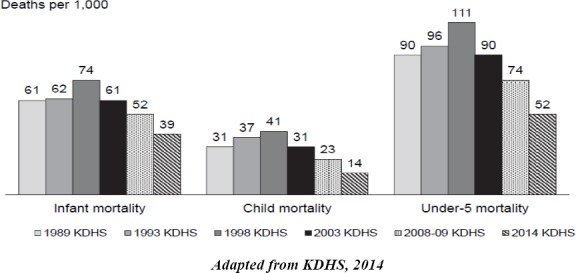
Trend in Child Mortality over years

Other initaivies reiterated were enhanced regulatory role of the government in health care provision; creation of an enabling environment for increased private sector as well as community involvement in service provision and financing; and increase and diversify per capita financial flows to the health sector agin key ingredients necessary in achieveing universal coverage. Two years later in 1996, the Ministry of Health with the support of key stakeholders developed the Kenya Health Policy Framework Implementation Action Plan, while at the same time established the Health Sector Reform Secretariat (HSRS) which was later transformed into a directorate in charge of health care financing at the Ministry to spearhead the implementation of the policies for purposes of coordinated planning and implementation. Similarly, a rationalization programme within the MOH was also initiated aimed at responding to the financing of the public health sector aimed at enhancing access to quality care amongst the poor and other vulnerables. Additionally, the National Hospital Insurance Fund Act was repealed in 1998 for purposes of enhancing financial protection and broadening the benefit package while enhancing governance of the institution. NHIF service coverage however, was not expanded at that time, while the benefit package remained narrow as discussed under sub-section 3. In the process, the populace continued to experience catasptrophic spending with low enrolment.

### 2.3 The Vision 2030

In 2008, the government launched the country’s development blue print dubbed “Vision 2030” with three pillars namely economic, political and social pillars. In the social pillar, health sector was accorded necessary priority since it was recognized that for the country to realize its set objectives, high quality of life was necessary (GoK, 2008). This was operationalized in the Medium Term Expenditure Framework (MTEF) of 2010 as well as the Health financing Strategy of 2010. In the MTEF, the central role of health as a key pillar was emphasized by the government commiting itself towards the provision of equitable and affordable healthcare at the highest affordable standard to the citizens. This was to be realized through the provision of robust health infrastructure including equipment, strengthening health service delivery, development of risk pooling financing mechanisms, while at the same time ensure AID effectiveness and harnessing social solidarity in the country through NHIF. This is further re-emphasized in the NHIF strategic plan of 2014 – 2018 (NHIF, 2014). Though the government through NHIF has continued to work towards this, more still need to be accomplished. For instance, revenue collections from the informal sector have remained insignificant, while the cost of collecting has continued to rise significantly with cases of fraud at facilities experienced (NHIF, 2015).

In the Health Financing Strategy, the government further committed itself towards universal coverage by emphasizing social health protection to all Kenyans by introducing social solidarity mechanisms founded on complementary principles of social health insurance and tax financing for purposes financial protection of the poor and other vulnerable groups. In order to achieve the set objectives, the government reiterated its intention of taking the necessary measures to amend the NHIF Act of 1998 for purposes of enhancing coverage among the poor, accelerating coverage of the informal sector, enhancing the benefit package, while ensuring improved governance and efficiency. While this is considered a bold step in the right direction, a formal assessment is yet to be undertaken to evaluate the impact.

### 2.4 The New Constitution and Health Bill, 2015

In the country, a new Constitution was promulgated in 2010 which among other issues provides the necessary legal framework for ensuring a comprehensive and people driven health care delivery aimed at enhancing access to quality health care. Specifically, the Constitution introduced a devolved system of governance with two tier government system – County and National government with the goal of enhancing utilization and geographical access to quality care by all Kenyansincluding the poor and other vulnerable groups. The constitution provides for the right to access health care including emergency health services by all including children and persons living with disabilities as key areas of focus in health services delivery.

A few years after the promulgation of the constitution, health services have been devolved as provided for in Schedule four (4) of the Constitution. There have however been increased concerns by the National government through the Ministry of Health to take over the health functions from the county governments. These according to the Ministry, has been necessitated by the near collapse of health care delivery at the county level. For instance, in majority of the counties, health workers have continuously been on strike sighting sloppy management of human resource and poor infrastructure. At one time the health services were paralyzed in a number of counties with women and children, among other vulnerable groups affected the most. Key concerns according to the health workers relate to poor health infrastructure, delays in salary in some counties up to five months, lack of schemes of services absence of the law upon which health services are to be implemented at County and National level, political interference, among others. These have partially contributed to increased utilization of private hospitals (faith based, clinics and hospitals) as reported in both print and mass media. Worse still, many of these facilities were at the time reluctant in admitting patients from under NHIF scheme sighting low capitation by NHIF and delays in payments. Given this scenario, although NHIF is seen as a way of enhancing social protection and, finally social solidarity, cases of household being at risk of financial catastrophe are likely to be experienced given poverty levels in many parts of the country.

Incidences of massive exodus of health workers, especially doctors from public health facilities, as well as dis-jointed purchasing of drugs and medical supplies, lack of policy guidelines have been witnessed in majority of the counties. If these issues are not addressed accordingly, the positive gains made may be affected negatively. Currently, the Health Act is the process being enacted however a few concerns have been raised regarding the Bill by professional associations. For instance, health professional associations, especially Kenya Medical Practitioners, Pharmacist and Dentists (KMPP&D) and Kenya National Union of Nurses KNUN) have voiced concerns about some provisions of the Bill. Their concerns range from scheme of service, discrepancies in salaries, with casual workers being paid higher salaries than qualified and employed doctors, lack of support for continuous medical education, a central body to address grievances, inability to define national referral system, regulation of agency, among others. All these issues unless addressed accordingly, have negative implications on quality of health services which in the process may impact negatively on the country’s realization of universal coverage. This is because a strong, efficient, well-run health system is a key ingredient towards UHC as envisaged by World Health Organization.

### 2.5 Recent Reforms and Initiatives

In 2013 during the Madaraka Day celebration, the government announced the abolishion of user fees at primary health care facilities and introduced free maternal health care services in public health facilities. This was lauded as a positive towards enhancing access to quality care especially the poor and other vulnerable groups. Whereas technically this is viewed as a positive move towards improving access to essential health services by the poor and other vulnerable, its implementation has been questioned by experts. The conecrens has been that the initiative lacked technical and necessary legal and operational policies. Technical input to inform the policy initiative is necessary otherwise this may instead undermine the intended objectives. Following the policy pronouncement, cases of delays in the disbursement of funds to counties have been common with a few opting for bank overdrafts to meet operational expenses not withstanding the embedded charges. As noted earlier, a system for financing health services is pivotal in UHC and if not carefully addressed, will negate the realization of UHC. Cases of stock outs of drugs and other medical supplies, poor maintenance of equipment, transport, and medical facilities are likely to be experienced in public health facilities.

Additionally, with the removal of user fees, media reports and in-depth interviews revealed increased utilization of health facilities which however led to cases of congestion, stock-outs, and break downs of machines in some facilities. Again, this raises equity issues in terms of financial protection and access to quality care. Recent initiatives by the First Lady of “*Zero tolerance*” campaign for expectant mothers, children and breast cancer are some of the latest efforts towards UHC. This has seen many stakeholders pull resources towards the initiatives although there are still no reliable statistics to inform policy dialogue. Whereas this is positive step and much lauded, there is lack of policy to support the initiative to ensure sustainability in the event of political regime change, which is undoubtedly expected in a democratic society like Kenya. It may be necessary to learn from society where such initiatives have been mooted and implemented like in the United States under the *Obama Care* initiative.

## 3. National Health Insurance Fund (NHIF) and Universal Coverage

**Figure 3:**
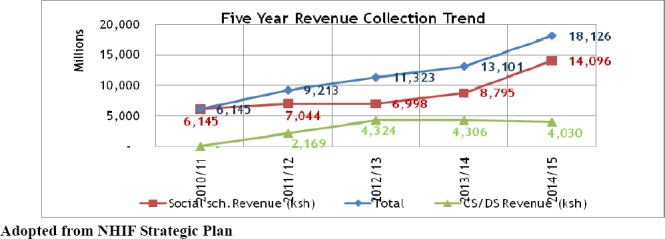
Revenue collection trend

In order to enhance access to health care as a step towards UHC, the government has identified and settled on NHIF as official vehicle for the successful implementation of universal health coverage for the country (NHIF, 2015). In this regard, there has been urgent need for NHIF to expand its benefits and coverage to other members of the society. Although the Act of 1998 provides for expansion of benefits, reports reveals low contributions which have not given NHIF the fiscal space to offer the intended benefits. Against this backdrop, the Government *gazetted* increased contribution rates in 2010 to cater for both in-patient and out-patient cover in an enhanced benefit package partially contributing to increament in revenue. Figure 3 provides a summary of these schemes since initiation in 2011.

**Figure 4:**
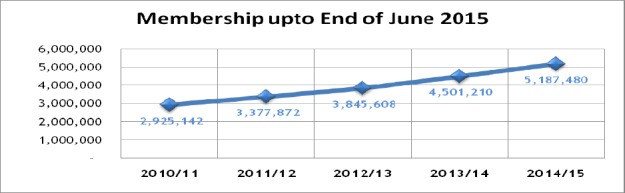
Membership Trends over the last five years

The national scheme envisages universal coverage in which both in patient and out-patient services for members are catered for. As such, three categories of membership have been identified as Formal, Informal and Indigents/Sponsored with monthly contributions rates (family cover) have also been varied with the formal sector being on a graduated scale ranging from Ksh.150 to Ksh. 1700. Informal sector contributions have been pegged at Ksh. 500, while the voluntary/sponsored category has been set at Ksh. 300 per month. In terms of membership, members are required to register with the scheme and declare their preferred facility of choice including their declared dependents for capitation purposes. As a starting point, access to out-patient services forms the entry point, while in patent services can only be provided on referral from an outpatient case, although some can occur on demand based on level of care requirements. This has contributed towards increase in the membership as summarized in Figure 4.

Regarding benefit package, the envisaged enhanced package includes in-patient, outpatient and maternity services, with the treatment protocols pegged on Kenya national treatment guidelines and the Kenya Essential Package for Health (KEPH). The package is available to both the principal member and declared dependants. Reports show that NHIF is currently engaged with private insurers to harmonize benefit package to avoid overlaps NHIF, 2015). The main challenge however, has been in adequate information for the general public on the scheme which seems to have had a negative impact on enrollment levels. However, facilities have also been up in arms on the amount NHIF has allocated as capitation currently pegged at Ksh. 1,200 although there are proposals to review the same upwards (NHIF, 2015). This could account to the pay-out ratios summarized in Table 2.

**Table 1:** Leading Causes of Deaths and Disabilities in Kenya

Causes of deaths				*Causes of DALY’s*		

Rank	Disease/injury	% total deaths		Rank	Disease/injury	% total deaths
1	HIV/AIDS	29.3		1	HIV/AIDS	24.2
2	Conditions arising during perinatal period	9.0		2	Conditions arising during perinatal period	10.7
3	Lower respiratory infections	8.1		3	Malaria	7.2
4	Tuberculosis	6.3		4	Lower respiratory infections	7.1
5	Diarrheal diseases	6.0		5	Diarrhoeal diseases	6.0
6	Malaria	5.8		6	Tuberculosis	4.8
7	Cerebrovascular disease	3.3		7	Road traffic accidents	2.0
8	Ischemic heart disease	2.8		8	Congenital anomalies	1.7
9	Road traffic accidents	1.9		9	Violence	1.6
10	Violence	1.6		10	Unipolar depressive disorders	1.5

Source: GoK, 2010; Draft Health Policy, 2012; Okech, 2014, 2012.

**Table 2:** The NHIF Social Scheme Pay Out Ratio for the last five years:

NHIF Payout Ratio(Social Scheme) FY 2010/11 - 2014/15			

Financial Year	Revenue (ksh)	Amount Paid	Pay out Ratio
2010/11	6,144,943,782	3,544,610,192	58%
2011/12	7,044,007,247	3,922,926,916	56%
2012/13	6,998,302,722	4,687,260,049	67%
2013/14	8,794,919,307	5,627,232,594	64%
2014/15	14,095,952,374	6,438,901,590	46%

Source: NHIF, 2015.

Whereas these initiatives are lauded for their positivity, there have been concerns on the accreditation process of facilities with reports indicating incidences of political interference riddled with political patronage, with facilities lacking necessary resources (human resources, infrastructure and equipment) being listed as preferred facilities. In this regard, equity in terms of quality care needs to be looked into. NHIF has also been reported to have continued to accumulate huge surpluses as shown in the pay out ratio. This has been primarily attributed to under-utilization, narrow benefit package, lack of incentives for public sector providers to seek reimbursements, high operational costs, and bureaucracy and poor governance issues (NHIF, 2015). Other weaknesses exhibited by NHIF include failure to clearly define the benefit package, limited products offered to the members, inadequate communication with the customers and other stakeholders, delays in receipting and posting of revenue especially that paid via EFT and RTGS, capitation method of payment is not well understood and accepted by both members and health care providers, lengthy accreditation process of healthcare provider, cases of fraud at the health care facilities, limited of provider payment mechanisms, among others.

## 4. Conclusion and Way Forward

### 4.1 Conclusion

There is considerable evidence that the Kenya government has been on *war* path towards universal health coverage, however there are equity issues that have riddled the whole process including quality of care, utilization and catastrophic spending by households especially the poor and other vulnerable. At the moment public providers face problems following the policy changes which unless carefully addressed will continue to affect the country’s desire to realize UHC. For instance, public health workers have continued to down their tools in most counties, sighting sloppy human resources management and dilapidated health infrastructure. Incidences of where patients continue to pay more for services than before continue to be reported with some reporting cases where they are asked to purchase drugs and other pharmaceutical supplies from private providers (chemist/pharmacies/drug stores) not withstanding their charges. On the positive side, the increased attendance of publicly provided primary health care services by the poor and reduction of financial risks as a cause for not using services provide a basis to argue that policy reforms continue to generate positive health outcomes and that the country is headed towards the intended objective. Lack of counterfactuals and rigorous controlled impact evaluation has however, in away impacted negatively on policy pronouncements with latest case of removal of user fees on lower level facilities and maternal health care proving a perfect illustration.

Despite the positive gains, Kenya’s progress towards universal coverage exhibit matters of concern like other developing countries. It appears that the display of leadership by the highest national authorities can be a sword with two edges. For instance, the country’s political leadership announced user fee removal policies for the public health sector out of the blue, without giving technocrats sufficient time to correctly design, prepare and implement the reform. Consequently, some national policies though considered politically sound, may fail to bring the expected. As observed by Jansen et al. (2010) as quoted by Meessen et al. (2011), public health includes three major fields: policy, practice and research. In the country, each of them is organized as an ecological niche. These are characterized by specific ideologies, norms, jargon, internal orientations, communication channels, internal codes of behavior, self-directed learning processes, autonomy and the desire to protect their way of functioning against the outside world (see Meessen et al., 2009; Harmonization for Health in Africa, 2010; Meessen et al., 2011). The disconnection between scientists - in charge of producing evidence), top officials (who have the required knowledge for policy making) and practitioners, who have the operational experience, largely explains the research-to-policy and the policy-to-implementation gaps: each party ignores or even despises the knowledge held by the other.

### 4.2 Way Forward

From the review, it is clear that Kenya government with support from its strategic partners has been on the forefront in accelerating the realization of universal health coverage. Notwithstanding this, the road has been bumpy thereby contributing towards health inequity at both micro and macro levels. Whereas devolution is expected to bring on board various positive gains, it is important that there is constructive engagement between the national government and county government and other key stakeholders on how to effectively deliver health care to the Kenyan populace. Key issues that merit attention will include fast tracking the enactment of Health Act currently under deliberation in parliament; increase investment in health care by considering mechanisms that embrass social solidarity; effieciency in allocation and utilization of the funds; reviewing and harmonizing the scheme of service across counties. Other recommendations include speeding up the process of reviewing the NHIF Act of 1998, to not only improve governance of the institution, but also improve its effectiveness and efficiency; review of the acreditation policy and strategy to foster credibility and objectivity; review the current benefit package to conform to the customer needs; support the initiatives of the First Lady on *beyond zero tolerant*, for purposes of sustainability; systematic planning and implementation of policies by involving all key stakeholders; continuous investment in human resources and health infrastructure development.
